# Antiatherosclerotic effects of licorice extract supplementation on hypercholesterolemic patients: decreased CIMT, reduced plasma lipid levels, and decreased blood pressure

**DOI:** 10.3402/fnr.v60.30830

**Published:** 2016-04-22

**Authors:** Yacov Fogelman, Diana Gaitini, Eli Carmeli

**Affiliations:** 1 Department of Family Practice, Leumit Health Services, Haifa, Israel; 2 The Ruth & Bruce Rappaport Faculty of Medicine, Technion – Israel Institute of Technology, Haifa, Israel; 3 Department of Medical Imaging, Unit of Ultrasound, Rambam Health Care Center, Haifa, Israel; 4 Department of Physical Therapy, Faculty of Social Welfare and Health Sciences, Haifa University, Haifa, Israel

**Keywords:** licorice extract, carotid intima media thickness, atherosclerosis, cardiovascular risk reduction, blood pressure

## Abstract

**Background:**

Ethanolic extract of licorice root has been shown to reduce low-density lipoprotein (LDL) oxidation in atherosclerotic mice and in both hypercholesterolemic and normal lipidemic humans.

**Objective:**

This study examined the effect of licorice-root extract on carotid intima-media thickness (CIMT) in individuals with hypercholesterolemia.

**Design:**

Individuals with hypercholesterolemia (total cholesterol ≥6.18 mmol/L [240 mg/dL]) and without significant stenosis were randomly allocated to two groups: an experimental group that consumed 0.2 g/day of ethanolic extract of licorice root for 12 months, and a control group that received a placebo.

**Results:**

Of 110 eligible participants, 94 (41–80 years old) completed the study. A significant CIMT decrease from 0.92±0.25 mm to 0.84±0.21 mm was observed in the experimental group compared with an increase from 0.85±0.17 mm to 0.88±0.19 mm in the control group. Mean plasma total cholesterol levels and LDL cholesterol decreased, at the range baseline to 1 year, from 284±32 mg/dl to 262±25 mg/dl and from 183±8.5 mg/dl to 174±9.1 mg/dl, respectively, for the experimental group (*p*<0.001) and from 291±35 to 289±31 mg/dl and from 177.6±10.7 to 179.3±9.6 (*p*=0.08), respectively, for the control group. Mean high-density lipoprotein (HDL) did not change significantly in either group. In the experimental group, systolic blood pressure decreased from 138±12 mmHg to 125±13 mmHg after 1 year (*p*=0.01) and increased from 136±15 mmHg to 137±13 mmHg in the control group. Diastolic blood pressure decreased from 92±9 mmHg to 84±10 mmHg (*p*=0.01) in the experimental group and increased from 89±11 mmHg to 90±8 mmHg in the control group.

**Conclusion:**

Following 1 year of licorice consumption, mean CIMT, total cholesterol, LDL levels, and blood pressure were decreased. This suggests that licorice may attenuate the development of atherosclerosis and of related cardiovascular diseases.

The dried roots of the licorice plant *Glycyrrhiza glabra* have been consumed for the past 6,000 years. They are used as flavoring and sweating agents, and as demulcents and expectorants in Western countries; and as anti-allergic and anti-inflammatory agents in Japan and China (1). Ethanolic extract of licorice root has been shown to reduce low-density lipoprotein (LDL) oxidation in atherosclerotic mice (2) and in hypercholesterolemic, as well as in normal lipidemic humans (2, 3). These and additional studies show that dietary consumption of licorice-root extract by hypercholesterolemic patients may act as an antioxidant agent against cardiovascular disease (4).

Carotid intima-media thickness (CIMT) is commonly used as a predictor of cardiovascular outcome in patients with metabolic conditions such as obesity, diabetes, and cardiovascular diseases (CVDs). The easy applicability and noninvasiveness of sonography render it suitable as a surrogate endpoint for measuring atherosclerotic burden in people with hypercholesterolemia risk factors (5).

The aim of this study was to analyze the anti-atherogenic effect of licorice-root extract consumption in patients diagnosed with hypercholesterolemia, according to changes in LDL and CIMT.

## Methods

### Study design

This is a randomized longitudinal cohort placebo-controlled study. Eligibility criteria were: total cholesterol ≥6.18 mmol/L (240 mg/dL) and the absence of moderate-to-severe stenosis, hypertension, diabetes mellitus, and ischemic heart disease. The participants, attending physician, and all those involved in the research were blinded from the group allocation. The main endpoint after 1 year was the change in CIMT, and the secondary endpoint was the change in lipid parameters.

### Patients

**Fig. 1:**
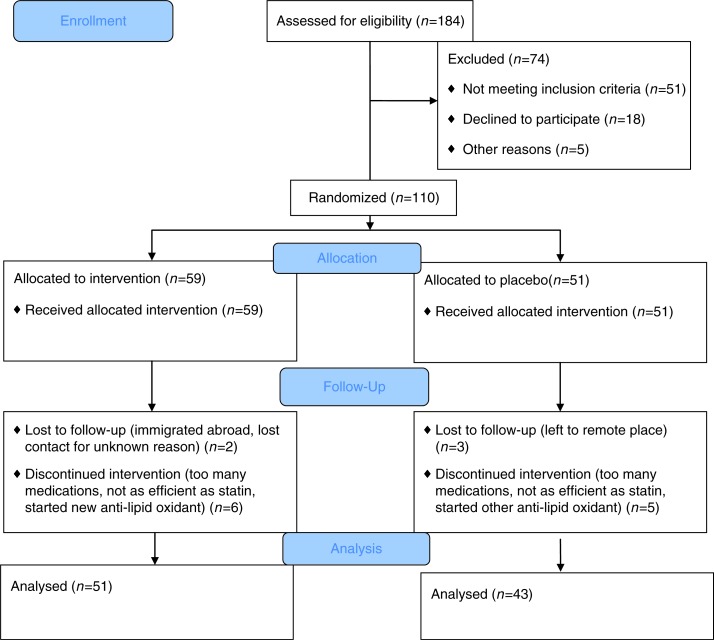
Consort 2010 flow diagram.

From a primary care clinic, we invited 184 patients to participate in the study. Of them, 51 did not meet inclusion criteria, 18 declined to participate, and 5 did not participate for other reasons (Fig. 1). The remaining 110 individuals were randomized and segregated into two study groups. In addition to blood lipid tests, all participants underwent a complete blood count and tests for fasting glucose, sodium, creatinine, and liver function tests (alanine aminotransferase, aspartate aminotransferase, alkaline phosphatase, gamma glutamyl transferase, bilirubin, and albumin), as well as a detailed medical history including smoking habits, physical examination, electrocardiography, and blood pressure measurements, as assessed by an average of 20 home measurements during 1 week. We included only participants not taking lipid-lowering drugs. Patients on any anti-lipid oxidant (e.g. vitamin E, omega 3, olive oil/fish formula, or juice supplement) were asked to stop taking it at least 2 weeks before consuming the licorice.

In a random number generator (using two playing cards: ‘A’ and ‘B’), the participants were allocated to two groups: experimental group (*n*=59) and control group (*n*=51). The experimental group consumed a daily dose of 0.2 g of DGL ethanol-extraction licorice (Fitness R Us LTD, Kiryat Shmona, Israel) for 12 consecutive months; the licorice capsule contained 4 mg of glabridin. However, the control group consumed a placebo drug. The color, odor, shape, taste, and packaging of the placebo were the same as that of the DGL ethanol-extraction licorice. The DGL ethanol-extraction licorice and placebo were supplied by the manufacturer free of charge; however, the manufacturer was not involved in the design and analysis of this study. Compliance was monitored by patients stating whether they missed taking any capsules, and by the collection of empty packages. The institutional ethics committee of Haifa University, which is under the administrative jurisdiction of the Israeli Ministry of Health, approved the study, and the protocol was implemented as planned.

### Outcome measures

CIMT was measured for all study participants before and one year after starting consumption of licorice or placebo. CIMT is defined as the distance between the luminal–intima and medial–adventitia interfaces at the distal 10 mm of the common carotid artery.

**Fig. 2:**
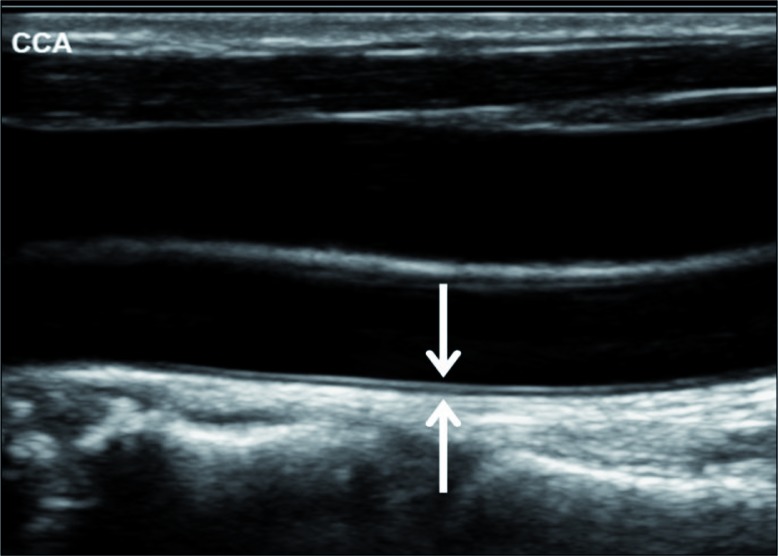
Intima–media thickness (IMT) is the blood–intima (upper arrow) to media–adventitia (lower arrow) interfaces width.

**Fig. 3:**
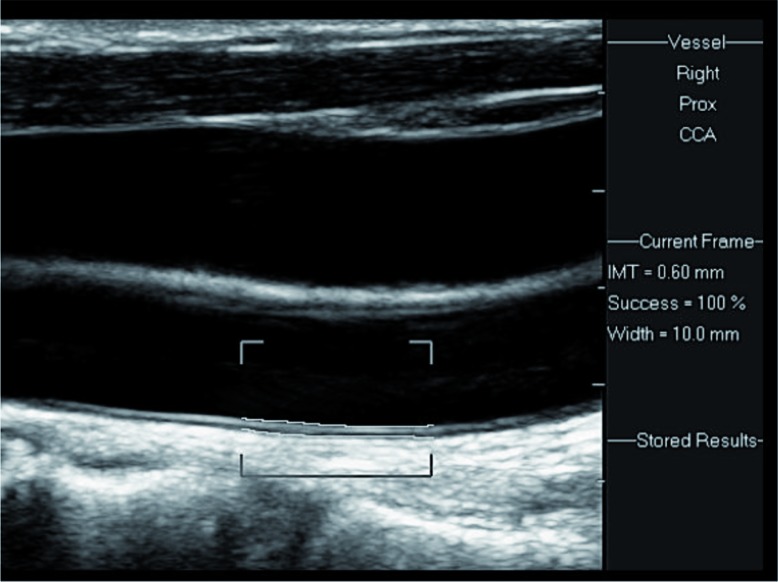
Electronic measurement of the IMT is performed by placing the cursors on the interfaces, along a centimeter at the distal common carotid artery. A normal IMT is demonstrated (0.6 mm width), with 100% success (see Success=100% at the side of the image).

**Fig. 4:**
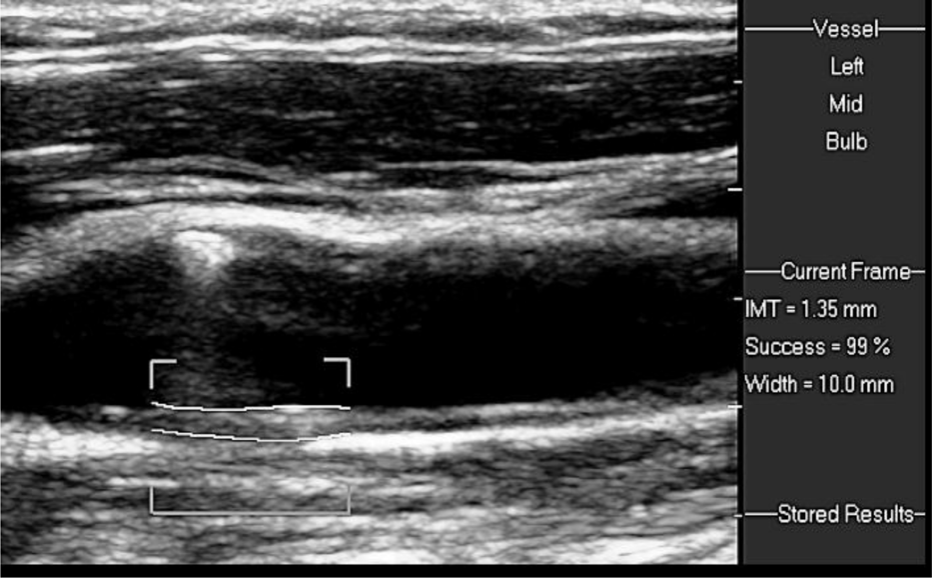
Moderately enlarged IMT. A 1.35 mm width on the distal CCA is measured, with 99% success.

Ultrasound examination of the carotid arteries was performed with patients in the supine position, with the head slightly hyper extended and turned to the opposite side. Axial and sagittal scans were obtained using a high-resolution linear transducer of 5–12 or 3–9 MHz (Philips IU22, Bothell, Washington). Wall thickness and atherosclerotic plaques in the carotid arteries were searched for. CIMT was measured on the far wall of the distal common carotid artery on each side, 1–2 cm proximal to the bifurcation (Fig. 2). Electronic software was applied, which follows the two echogenic lines on the far wall of the carotid artery: the lumen–intima interface and the media–adventitia interface (Fig. 3). Normal CIMT in adults is in the range of 0.6–0.8 mm. Slightly increased, moderately increased, and severely increased CIMT are in the ranges of 0.8–1.0, 1.0–1.5, and above 1.5 mm, respectively (Fig. 4). CIMT measurements were performed according to an international consensus statement [5], by skilled personnel working in the US Unit, Department of Medical Imaging.

### Categorization of the outcome measures

Progression and regression in carotid atherosclerosis were defined as an increase and decrease, respectively, in the CIMT from baseline to 12 months, following daily licorice or placebo consumption. The change was measured in the same pre-selected carotid artery segments.

### Statistical analysis

All analyses were carried out with the SPSS Version 21 statistical package. Quantitative results were reported as mean±SD. The data were tested by analysis of variance (ANOVA), and the means were compared across groups by Tukey's test; significance was considered as *p*≤0.05.

## Results

Of the 110 individuals who were randomized to experimental and placebo groups, 94 concluded the study. Sixteen participants, eight from each group, dropped out of the study. None of the participants stated that they dropped out due to intolerability of the drug. All 94 participants stated that they consumed the licorice supplement or the placebo for at least 90% of the time throughout 365 days. Fifty-one participated in the experimental group (24 men), age ranging from 41 to 80 years; mean age is 61.5±16.3 years. In the control group, 43 individuals participated (21 men); age ranged from 45 to 77 years, and mean age is 60.4±15.7. The mean time between the first and the second assessment was 11.9±1.2 months. Mean CIMT was 0.92±0.25 mm at baseline in the experimental group and was significantly lower at the second assessment (0.84±0.21 mm, *p*=0.000). In the control group, mean CIMT at baseline was 0.85±0.17 and was significantly higher at the second assessment (88±0.19). Clinical characteristics of the study groups are presented in Table 1.

**Table 1:** Clinical characteristics of the patients in the experimental (*n*=51) and control group (*n*=43)

	Experimental group			Control group		
		
	Baseline	After 1 year	Sig. (two-tailed)	Baseline	After 1 year	Sig. (two-tailed)
BMI (kg/m^2^)	28.3±3.07	28.2±3.12	NS	28.1±2.8	27.9±2.9	NS
Mean CIMT (mm)	0.92±0.25	0.84±0.21	0.000	0.85±0.17	0.88±0.19	0.000
LDL (mg/dl)	183±8.5	174±9.1	0.000	177.6±10.7	179.3±9.6	0.08
HDL (mg/dl)	39.8±9.2	39.9±9.4	NS	38.6±8.9	38.9±8.1	NS
TC (mg/dl)	284±32	262±25	0.000	291±35	289±31	0.06
Systolic BP (mmHg)	138±12	125±13	0.01	136±15	137±13	NS
Diastolic BP (mmHg)	92±9	84±10	0.01	89±11	90±8	NS
Smokers	9	9		7	6	

None of the baseline lipid parameters measured (total cholesterol, LDL-cholesterol, HDL-cholesterol) was significantly correlated with changes in CIMT (Pearson correlation 0.338 for LDL-C). Associations between CIMT, and age and BMI could not be determined. However, CIMT was not found to be associated with blood pressure (Pearson's correlation=0.738).

We assessed for both study groups the 10-year risk of fatal CVD death by SCORE evaluation: cardiovascular risk charts based on gender, age, total cholesterol, systolic blood pressure, and smoking status, with relative risk chart (www.escardio.org/static_file/Escardio/Subspecialty/EACPR/Documents/score-charts.pdf).

In the experimental group, the 10-year relative risk decreased from 11.2±3.6% at baseline to 9.4±2.9% after 12 months (*p*<0.05), compared to a slight increase in the control group from 10.8±3.1% to 10.9±3.3%, respectively (*p*=NS).

## Discussion

CVDs are leading causes of death and disability worldwide. The role of micronutrients has been studied extensively as a CVD risk minimizing intervention. These include antioxidants that are available over the counter as dietary supplements. Despite high commercialization of such products, scientific evidence and clinical trials supporting their use are not conclusive (6). The lipid per oxidative hypothesis of atherosclerosis indicates that LDL oxidation plays a crucial role in the early stage of atherogenesis (7). This hypothesis is supported by evidence that oxidized LDL is present in atherosclerotic lesions (8) and in human plasma from patients with CVDs, and that it correlates with the presence of angiographically documented complicated plaques in mice (9). Antioxidants can protect LDL from oxidation not only by their binding to the lipoprotein, but also following their accumulation in cells of the arterial wall (10).

Licorice was shown to be a very potent antioxidant against LDL oxidation (11) and, in parallel, to inhibit atherosclerosis development in mice deficient in vitamin E (12, 13). One-month consumption of ethanolic extract of licorice root by moderately hypercholesterolemic patients was shown to reduce plasma cholesterol levels by 5%, plasma LDL levels by 9%, and blood pressure by 10% (4). These results are similar to those of our study. However, our results were recorded after 1-year consumption.

In clinical practice, risk prediction algorithms have been used widely to identify individuals at high risk for developing CVD in the short term to select individuals for more intensive preventive interventions. In this study, we used the SCORE algorithm, derived from European data, to evaluate a risk score that predicts fatal cardiovascular events. The risk reduction in the experimental group is probably due to total cholesterol and blood pressure reduction.

The relationship between licorice consumption and CVDs may be due to attenuation of LDL oxidation, macrophage foam cell formation, and progression of atherosclerosis. The effect of licorice on arterial cell-mediated oxidation of LDL is determined by its accumulation in the lipoprotein and in arterial cells, such as macrophages. Licorice can reduce LDL lipid peroxidation by scavenging reactive oxygen/nitrogen species, chelation of transition metal ions and sparing of LDL-associated antioxidants. Licorice can also reduce oxidative stress of macrophages by inhibition of cellular oxygenases (12, 14). Oxidized LDL is highly atherogenic as it stimulates macrophage cholesterol accumulation and foam cell formation; it is cytotoxic to cells of the arterial wall and stimulates inflammatory and thrombotic processes. LDL oxidation can lead to subsequent aggregation, which further increases cellular cholesterol accumulation (15).

Recent improvements in imaging technology have made possible noninvasive assessment and identification of early vascular changes using ultrasonography. B-mode ultrasound is a noninvasive, accurate modality that accurately visualizes the arterial wall thickness. CIMT is constituted by the combined thickness of the intima and media layers of the artery wall, measured at the far wall of the distal common carotid arteries. CIMT is measured by high-resolution B-mode ultrasound of extra-cranial carotid arteries and is the most widely accepted and the only non-invasive surrogate marker for early atherosclerotic disease (16–18). Increased CIMT is an intermediate stage in the continuum of atherosclerosis, which significantly correlates with coronary and cerebrovascular disease (19). Epidemiological studies have consistently reported a predictive value of increased CIMT for myocardial infarction and stroke, independent of traditional CV factors (20, 21). This has been confirmed in a meta-analysis of 12 relevant general population-based studies (22). The current study showed a noteworthy decrease in CIMT in the experimental group compared with the control group. Pomegranate juice consumption was also shown to lead to significant CIMT reduction, by up to 30% after 1 year. The effect was attributed to the potent antioxidant characteristics of pomegranate juice polyphenols (17). Elevated CIMT in patients with cardiovascular risk factors, such as diabetes mellitus, hypertension, smoking, male sex, and advanced age is associated with an increased risk for myocardial infarction and stroke. A decrease in CIMT subsequent to drug treatment is associated with reduced incidence of vascular events (18, 19).

We did not find a statistically significant association between CIMT and blood pressure. However, other studies have found such associations. CIMT was found to be associated with isolated systolic hypertension in asymptomatic elderly subjects (23), with hypertension in individuals with rheumatoid arthritis (24), and with systolic blood pressure among women treated for hypertension (25). We found that LDL-cholesterol at baseline and after 1 year was not associated with changes in CIMT. In a recent prospective study, changes in CIMT were only predicted by the proportion of sdLDL (small density particles) but not by LDL-C levels (26).

Several explanations may be proposed for our findings, including a number of mechanisms of antioxidant activity against LDL oxidation, and the pathogenesis of atherosclerosis prior to clinical presentation. Therefore, we suggest that future study should focus on investigating the biological mechanism of licorice, such as its role in oxidative damage/scavenger clearance and in preventing accumulation of free radicals due to lipid peroxidation. Elucidation of the biological mechanism may lead to a more effective strategy for the treatment of anti-atherosclerotic phenomenon in patients with hypercholesterolemia.

The strength of this study is its prospective longitudinal character that enabled investigation of a well-defined cohort of patients. Furthermore, the single-center design ensured uniform assessment of clinical and biochemical parameters, at the same laboratories and by the same investigators, therefore avoiding interobserver biases. This is of particular importance with respect to the measurements of intima media thickness. A limitation is the relatively small sample size. Furthermore, we did not measure sdLDL and flow mediated dilation (FMD), which can detect functional changes in blood vessels. While mean CIMT decreased significantly in the experimental group and increased somewhat in the control group, the lower mean baseline CIMT in the control group compared with the experimental group raises the possibility of selection bias.

In conclusion, this study demonstrated possible anti-atherogenic capabilities of licorice in three related components of atherosclerosis: plasma lipoproteins, blood pressure, and arterial CIMT. We suggest that a potent anti-oxidative capacity of licorice against lipid peroxidation may be the central link for the anti-atherogenic effects of licorice.
